# International selection and competition in youth sport: pin the tail on the donkey or targeted intervention?

**DOI:** 10.3389/fspor.2023.1298909

**Published:** 2023-12-21

**Authors:** Liam Sweeney, Áine MacNamara, Jamie Taylor

**Affiliations:** ^1^ Department of Sport Science and Nutrition, Faculty of Science and Engineering, Maynooth University, Kildare, Ireland; ^2^ School of Health and Human Performance, Faculty of Science and Health, Dublin City University, Dublin, Ireland; ^3^ Insight SFI Centre for Data Analytics, Dublin City University, Dublin, Ireland; ^4^ Grey Matters Performance Ltd., Stratford Upon Avon, United Kingdom

**Keywords:** talent development, talent identification, sport performance, youth sport, sports coaching

## Abstract

Across sporting contexts, there is growing debate surrounding the utility of junior international age group selection in sport. In this regard, there has been considerable focus on the age of selection, with the low conversion of athletes from junior to senior international level often used to critique the efficiency of such programmes. In this perspective article, we argue that there is a need for a more nuanced consideration of the effectiveness of international age groups in talent systems. We begin this perspective article with a synthesis of the literature pertaining to junior to senior transitions in sport, followed by the implications and opportunity cost presented by international age groups. We argue for a more contextual evaluation of international age groups relative to the performance aims of a talent system, the need for manipulation of challenge dynamics, and the resource costs of doing so (e.g., providing developmental challenges for those who have early advantage, or spreading resource amongst greater numbers for broader impact). We suggest that talent systems evaluate the opportunity cost presented by international age groups, with decisions based upon individual strategic context.

## Introduction

In the competitive landscape of high-performance sport, there is significant pressure for talent systems to select and develop athletes to the elite senior standard (1). Reflecting this, under the guidance of national organisations, thousands of young athletes across sports are selected each year to engage in Talent Development (TD) systems (e.g., academies of professional sports clubs, regional training camps, provincial TD squads) that are often well resourced relative to community participation (2). Those selected typically receive professional coaching, sports science and medical support, access to superior training equipment and facilities, and are exposed to high levels of competitive challenge compared to non-selected peers (3). One reason for the allocation of these resources and support systems is the provision of optimal challenge and opportunities to facilitate long-term development. Further to selection for talent systems, many national organisations utilise another layer of TD provision in the form of junior international age group squads. For example, some national soccer associations implement junior international age groups from as young as fourteen years of age, whereby players are exposed to regular training and international competition (4, 5).

The effectiveness of early selection, however, has consistently been questioned across the literature (6), especially when the range of biopsychosocial interactions that impact a young athlete's progression are considered (7). Further, given that athletes follow individualised, unpredictable, and typically non-linear developmental trajectories (6), there is a need to practically consider the objectives of junior international age groups, set against the considerable research base showing low conversion rates from junior to senior international level across sports (8–16). This perspective piece will critically consider implications of this research, making recommendations both for research and practice.

## Athlete conversion from junior to senior sport

A recent systematic review examining the conversion of youth to senior international-level athletes in sports including athletics, cycling, swimming, tennis, basketball, handball, rugby, and soccer concluded that “successful juniors and successful seniors are largely two disparate populations” (16). This analysis of studies with combined samples of over 60,000 athletes found that 89.2% of international-level U17/18 juniors failed to reach the international-level as seniors and 82% of international-level seniors were not selected for the U17/18 junior international level (16). In a similar investigation, Güllich (8) identified an average annual turnover rate of 24.5% at the academy level and 41% at the national level in German U10-U19 soccer, with only 7% of players identified at the U10 age group remaining within the system at the U19 age group. Herrebrøden and Bjørndal (13) explored the link between selection at the U17, U19 and U21 international level and subsequent elite experience (international appearances at the senior level and senior appearances at club Champions League and/or Europa League level) in 1,482 soccer players across Sweden, Denmark, Norway, Germany, Belgium, and Portugal. Whilst participation at the U21 level was correlated with senior elite participation, U17 participation was either an insignificant, or negative predictor, of progression to senior international soccer. Players at the U17 level were also less likely to gain experience at U19 and U21 levels than those with no U17 experience (13). Schroepf and Lames (9) examined the career patterns of 636 national team soccer players born between 1987 and 1994 who represented Germany at any stage from U16 through to the senior international team, identifying a total dropout rate of 73.5% from the U16-U21 squads, with most players' (38%) national youth team careers lasting only one year (9). Boccia et al. (11) note that less than 20% of senior international Italian soccer players selected between 2000 and 2021 represented Italy at the U16 level. In short, a low conversion of athletes from junior international selection to the elite senior level has consistently been observed across sporting contexts [e.g., (10–12, 14, 15, 17)].

In practice and research, the conversion of athletes from the junior to senior level has become a marker to quantify effective practice. For example, the Australian Institute of Sport refer to the need for “sporting organisations [to] strive to improve the conversion rates from national representation to podium and onto sustained success” (18). Yet, there is growing recognition that the vast majority of early selected youth athletes are unlikely to progress and become elite senior athletes (16). Indeed, the consistently low conversion rates across junior international age groups have been used to suggest that NGBs should explore alternative talent identification and development processes (11). Yet, conversion of junior athletes to senior levels cannot be viewed in absolute terms. For example, Schroepf and Lames (9) identified that there were places available for 636 youth soccer players born between 1987 and 1994 for a German youth international team (U16–U21), but only 37 players born between 1987 and 1994 made an appearance in the senior international team. Thus, the maximum conversion rate from the junior to senior international level for athletes across those seven age groups was only ∼6%. Interestingly, conversion rates of 9–15% from the junior international to senior international level have been found in other Football Associations, yet have been described as evidence that youth international performance is poorly correlated with senior performance (11). Thus, to judge the effectiveness of a TD system based on conversion rates is too simplistic and not reflective of the limited spots available at the highest levels of international sport, relative to those available in junior international age group squads (i.e., a squad per one-two year age group), and the pyramid design that is typical of TD pathways (19, 20).

Instead, there is a need for broader consideration and contextualisation of this type of data. In this regard, Taylor et al. (21) used the Performance Outcome Process [POP (22);] structure to consider levels of effective practice in TD systems. As an output measure of effectiveness, ultimate “performance” can be judged by the talent system's overall progression of athletes to the highest level, whilst also considering the extent to which athletes are prepared for life outside of sport (23, 24), their physical, psychological and social development and perceived quality of life (25, 26), and the system's impact on broader participation (27, 28). In contrast to effectiveness, system efficiency is related to resource allocation based on the limitations of all talent systems in terms of finance, time and attention (21). As such, conversion from junior to senior level is better seen as a marker of efficiency, rather than effectiveness. It is in this context whereby the investment of significant resources into a minority of youth athletes at the international level in preparation for senior international performance should be viewed and strategic effectiveness considered.

## Considerations

Whilst international age group selection may allow for the provision of focused and high-quality developmental input to those *more* likely to progress (29), those selected for youth international squads still only represent a minute proportion of the total participant population. Indeed, Güllich (8) estimate that players nominated for youth international soccer teams in Germany account for only 0.06% of all registered players within their respective age categories. Youth international squads are likely to be relatively resource intensive, with individualised athlete support through high quality coaching, sports science, medical provision, and international travel and competition (5, 16). Given that the vast majority of those selected for youth international squads will not become senior internationals (8, 9, 11, 13, 15, 16), it has been suggested that resources may be better invested in a broader population at the community level promoting recruitment, participation and development (13). Güllich et al. (16) made a similar argument, noting that such resources may be better spent when reallocated, particularly as most senior international athletes do not participate in international squads as junior athletes. In line with this, Erikstad et al. (28) pointed to the alternative strategy of investing in a broader population to enhance opportunities for developmental sporting experience rather than investing in a very select few. In short, all authors supporting previous calls for an emphasis on development and inclusion being a core feature of sporting systems (19).

In terms of systemic considerations, another risk factor is that selection to a youth international team will add another environment requiring integration into the pathway of the athlete; a current challenge across TD systems (30–33). Integration being the extent to which systems and stakeholders work in tandem to support the athlete, vertically and horizontally (31). An example of this lack of integration being the exposure of youth athletes to excessively high and unmanaged training volumes across their pathway, increasing the risk of burnout and injury (30, 34). Similarly, a lack of integration can leave athletes unable to make sense of their international-level experiences to best promote their own development (35).

For those selected for a youth international squad, there is also a need to consider the risks to athletic identity and status conferred by early selection (26, 36). Young athletes selected into formalised TD programmes are those at the highest risk of an exclusively athletic identity (26). Moreover, early selection into such TD systems and the early status acquired may also provide a level of validation and a reduced performance expectation that may lead to reductions in the perceptions of challenge, as well as reductions in commitment and losses in motivation (31, 37). These factors are significant derailers in the development of those high performing youth athletes who do not progress to the elite senior level (37).

It is also important to recognise the contextual differences when evaluating youth international selection and competition. For example, the average age of peak international performance in sports like gymnastics or diving occurs in early adulthood (38), and in some particular gymnastic events, athletes compete at the elite senior level in adolescence (39). In diving and skateboarding, senior Olympic medals have been won by athletes as young as thirteen years old (40). In such instances, international age group selection may offer an opportunity to provide the developmental provision necessary to prepare athletes for senior international competition within a relatively short timeframe. In other instances, such as soccer, triathlon, rowing, tennis or cycling, peak international performance may not occur until late twenties or early thirties (38). As such, the time delay between junior international selection and senior status may be a contributing factor in some sports.

## Challenge dynamics

Whilst early selection and superior performance outcomes as a junior do not necessarily mean one will be successful at the senior level, the most successful youth athletes are *more likely* to progress to elite senior status than their age matched less successful peers (29). For example, an analysis of 67,600 junior (U18 and U20) and senior athletes from track and field athletics demonstrated that those junior athletes ranked in the top 50 at the U18 and U20 level were significantly more likely reach the elite senior level when compared with less successful junior athletes at these respective age groups (29). For this reason, participating in international age group teams has been suggested to have a significant impact on later participation in professional and international sport (41). For example, 31% of Bundesliga players (top two divisions in German professional soccer) between 2009/10 and 2011/12 had played at least one match in a youth international team (8). Similarly, research from Portuguese international soccer demonstrates that of those youth players selected into a junior international team at ≤16 years, 34% progressed and made at least one appearance for the senior international team (42). When considering those selected for the youth international team between the ages of 17–18 years, 62% made at least one appearance for the senior international team (42). Similar research from Norwegian handball suggests that senior international players had accumulated more hours in youth international team training and competition than non-elite senior players (43). Of those youth athletes who competed in the 2010 and 2012 World Junior Athletics Championships, 73.8% and 71%, respectively, progressed to the (senior) International Association of Athletics Federations (IAAF) rankings within two years and 20.4% of World Junior Championship athletes from 2002 to 2012 achieved top ten (senior) IAAF rankings within an average of 2.4 years (44). Thus, whilst the conversion of youth athletes to the senior international level might appear low, engagement in youth international team activities may be an important feature in the development of some senior international-level athletes (35).

Whilst there is the potential for inappropriately early recognition and validation, early selection does provide the opportunity for the appropriate orchestration of challenge dynamics; challenge defined as a memorable experience that a performer perceives to disrupt their development and/or performance in sport (35, 37, 45–48). Challenge dynamics are, therefore, the complex biopsychosocial factors that influence an individual's experience of and interaction with challenge (49). In education, this need for challenge has been referred to as asking people to “go beyond a safety zone…and venture out into possibly dangerous and risky areas” (50). This orchestration of challenge dynamics may be especially relevant for early high performing athletes whose previous experience of their sport is likely to have been challenge-less compared to age matched peers. Crucially, without the acquisition and development of an adequate psychological skillset that challenges can test, athletes can be derailed by step changes in challenge that can occur towards the higher echelons of performance (37). In contrast, if a range of appropriate challenges can test the development of psycho-behavioural skills, the consequent emotional disturbance, and appropriate support, can encourage refinement of these skills (51).

Low conversion rates from international youth to international senior athlete notwithstanding (8, 11, 13, 15, 16), providing relatively early high performing athletes with appropriate levels of challenge might be essential if they are to progress (37, 52). Indeed, a lack of challenge significantly inhibits the development of relatively early high performing youth athletes (35, 37, 52). For athletes with significant early advantages relative to peers, international-level competition may be the only environment perceived as challenging enough to provoke emotional disturbance and subsequent periods of reflection. In this regard, how can those relatively early high performing athletes be provided with the appropriate levels of challenge that appear essential in facilitating their long-term progression?

Whilst the low conversion rates are cited as an argument against the efficiency of international age groups (e.g., (10, 11, 17), early selection and a consistent athlete turnover also enables a larger pool of athletes to receive international-level experience (8). From a systemic perspective, this is proposed as beneficial as it allows national systems to monitor a large group of players and minimise the frequency of successful seniors developing outside the national system (8). At the individual level, participation in international age groups can offer unique life experience and learning opportunities that alternate contexts cannot provide (16), alongside the challenging experiences necessary for long-term development (35).

## Implications

In a world with limited resourcing, it is practically difficult to offer appropriate and individualised challenge to the large population of young athletes that participate in sport (21). From a practical perspective, international age groups offer the opportunity for talent systems to focus their limited developmental resources on those *more* likely to become high-level senior performers (29). There are, however, a range of considerations for the practitioner. In terms of efficiency, the early identification and subsequent transition of athletes in terms of conversion may be a marker of efficient practice. Yet, there also needs to be a consideration of effectiveness. Are the highest potential athletes actually being selected? What is the impact of higher resource allocation for the few on broader development? Can the needs of relatively high early achievers be catered for without selection practices? What are the impacts of patterns of selection and deselection more broadly? Here we argue that there are no simple answers to these questions, but there is a need for a more practically grounded and careful consideration of how international age groups are used. This should not only be in terms of the research that considers the evidence for particular strategic directions in TD, but also for practitioners who require an understanding of the strategic imperatives of different programmes. This is in essence a consideration of the relative costs of either not using international age groups, set against the costs of overly resourcing a limited number of early high performers who may, or may not be more likely to progress than peers outside of formal TD programmes. Ultimately, how organisations use international age groups is unique to their individual strategic context. For instance, some national organisations use international age groups to prepare selected athletes for future international competition (5), whereas others use international age groups as an opportunity to expose a large population to international-level competition and to minimise the frequency of successful senior athletes developing outside of the national system (8).

Whilst there are a number of considerations for the practitioner based upon their individual strategic context, we argue that the use of conversion rates as a measure of effective practice across systems is inappropriate. Indeed, it could be argued that in some systems they become a perverse incentive and a barrier to effective practice in the long term. Instead, selection might be viewed as an opportunity to provide additional support for early achievers and an appropriate level of challenge for those whose early experience of their sport is disproportionately easy (37). This, however, needs to be coupled with the use of multiple selection and deselection routes to ensure opportunity for those not subject to a range of early advantages. This suggests a novel framing of selection and may help to navigate some of the difficulties of player labelling and early identification (53). As such, we suggest that TD systems need to recognise the opportunity cost presented by international age groups and make decisions based upon their strategic context.

How and why talent systems structure their international age group activities should be a matter of strategic intent, based upon clarity of POP structure, rather than a perception of best practice (21). A shared understanding of the purpose of such initiatives is likely to enhance overall integration and, therefore, enhance their effectiveness (21, 31). In practical terms, this understanding should be based on the purpose and function of international age groups, supported by the integration of systems and support figures to maintain coherence for the athlete (31).

## Conclusion

So, are junior international age groups a targeted intervention or simply a game of pin the tail on the donkey? In this perspective article, we have argued for a multi-dimensional view of junior international age groups, early selection, and the relative resourcing of individuals at different stages, relative to each individual talent system. A significant volume of research converges on the typical non-linearity of development. Practice and research now needs to look at considerations for practice at all levels of the talent system. Whilst the implementation of international age groups presents an opportunity to focus developmental resources on those young athletes who may be *more* likely to progress, should this be at the detriment of providing appropriate resources and opportunities to the majority of young athletes? Likewise, can a talent system provide an equity of developmental provision across a population of young athletes, and yet still appropriately challenge the minority of exceptionally high early performing young athletes? This contextual consideration should be relative to the performance goals of a talent system (development for the elite level, or broader impact), the relative need for modulation of challenge dynamics and the resource costs of doing so.
